# Solvent-controlled growth of inorganic perovskite films in dry environment for efficient and stable solar cells

**DOI:** 10.1038/s41467-018-04636-4

**Published:** 2018-06-08

**Authors:** Pengyang Wang, Xingwang Zhang, Yuqin Zhou, Qi Jiang, Qiufeng Ye, Zema Chu, Xingxing Li, Xiaolei Yang, Zhigang Yin, Jingbi You

**Affiliations:** 1 Key Laboratory of Semiconductor Materials Science, Institute of Semiconductors, Chinese Academy of Sciences, Beijing, 100083 China; 2 College of Materials Science and Opto-electronic Technology, University of Chinese Academy of Sciences, Beijing, 100049 China

## Abstract

Inorganic halide perovskites such as cesium lead halide are promising due to their excellent thermal stability. Cesium lead iodide (CsPbI_3_) has a bandgap of 1.73 eV and is very suitable for making efficient tandem solar cells, either with low-bandgap perovskite or silicon. However, the phase instability of CsPbI_3_ is hindering the further optimization of device performance. Here, we show that high quality and stable α-phase CsPbI_3_ film is obtained via solvent-controlled growth of the precursor film in a dry environment. A 15.7% power conversion efficiency of CsPbI_3_ solar cells is achieved, which is the highest efficiency reported for inorganic perovskite solar cells up to now. And more importantly, the devices can tolerate continuous light soaking for more than 500 h without efficiency drop.

## Abstract (web summary)

Cesium lead iodide inorganic perovskite solar cells have great potential but the phase instability hinders their development. Here Wang et al. show a controlled drying process to make phase stable and highly efficient solar cells with power conversion efficiency of 15.7%.

## Introduction

Halide perovskite semiconductors used in photovoltaic devices were reported by Miyasaka et al. in 2009. The power conversion efficiency (PCE) has increased rapidly to more than 20% in the past several years^1–14^. And the large area module of perovskite solar cells was also demonstrated recently^15^. Traditional perovskite solar cells were mainly based on organic–inorganic hybrid materials such as methylammonium lead halide (MAPbX_3_, X = Cl, Br, I) and formamidinium lead halide (FAPbX_3_, X = Cl, Br, I), or their mixture^1–14^. In addition to humidity instability, the organic–inorganic hybrid perovskite materials also suffer from poor thermal stability due to easy evaporation of the organic parts^16, 17^.

Inorganic halide perovskite (CsPbX_3_) (X = Cl, Br, I) could be more thermally stable, it was found that CsPbX_3_ can sustain temperatures exceeding 400 °C without any phase degradation^18, 19^. This could be the reason of the significant photostability improvement of perovskite solar cells, while incorporating inorganic metal cations such as Cs or Rb into the organic cations in MAPbI_3_ or FAPbI_3_ perovskites^11, 20–23^. In addition to the excellent thermal stability, inorganic perovskite such as CsPbI_3_ showed an optical bandgap of 1.73 eV^24, 25^, which is an ideal material to configure tandem cells combined with either silicon or low-bandgap perovskite^26–30^. Even though similar bandgap could also be obtained from organic/inorganic perovskite by mixture halide strategy, halide segregation could be a critical issue for affecting photostability of the devices, while the halide dopant is more than 30% (such as FAPbI_0.6_Br_0.4_)^27^. Therefore, it is very meaningful to achieve high efficiency and stable CsPbI_3_ solar cells. However, it was found that the α-phase (black phase) of CsPbI_3_ could be rapidly degraded to non-photoactive δ-phase (yellow phase) in an ambient environment with moisture^25, 31–35^. It has been explained that the moisture can effectively introduce vacancies in the crystal lattice and lower the free-energy barrier to nucleation, and trigger the phase transition of CsPbI_3_ perovskite even at room temperature^32, 33^.

Previously, there are several efforts to stabilize the α-phase of CsPbI_3_ to make efficient solar cells^25, 34–42^, such as tuning the tolerance factor of perovskite structure by partially substituting iodide with bromide to form CsPbI_2_Br or CsPbIBr_2_^34–37^, reducing the crystal size^25, 40, 42^, or introducing intermediate phase such as Cs_4_PbI_6_^41^. All these efforts push the efficiency of inorganic perovskite solar cells to around 10%. Recently, during the preparation of this manuscript, significant progresses were witnessed, around 13% PCE of CsPbI_3_-based solar cells were reported by either doping B site in ABX_3_ perovskite structure^43^ or by passivating/stabilizing CsPbI_3_ quantum dot colloid via organic salt molecular^44, 45^. Even though, there is still a large room for further improving the PCE of CsPbI_3_ solar cells. To deliver higher efficiency of CsPbI_3_-based perovskite solar cells, two issues must be resolved. One is forming stable α-phase of CsPbI_3_ films^34^. Another one is obtaining the high quality of CsPbI_3_ layer, similar to the initial development of organic–inorganic perovskite solar cells, the pinholes, and grain boundary in the active layer usually leading to serious recombination and also poor device performance^46–48^.

Herein, we show a simple solvent-controlled growth (SCG) method to produce high-quality α-phase CsPbI_3_ perovskite thin films. To avoid phase transformation of CsPbI_3_ films, from black to yellow phase triggered by moisture^25, 31–33^, we processed the films in dry nitrogen environment, and stable α-phase of CsPbI_3_ was obtained. Adopting the high quality and stable α-phase of CsPbI_3_ as absorption layer to configure the solar cells, we achieved a PCE of 15.7% and a certificated PCE of 14.67%, which represent the highest level of inorganic perovskite solar cells so far. More importantly, our preliminary results show that the CsPbI_3_ solar cells own excellent photostability, the device can tolerate more than 500 h of continuous light soaking, and no significant efficiency drop is observed.

## Results

### Growth of CsPbI_3_ films

**Fig. 1:**
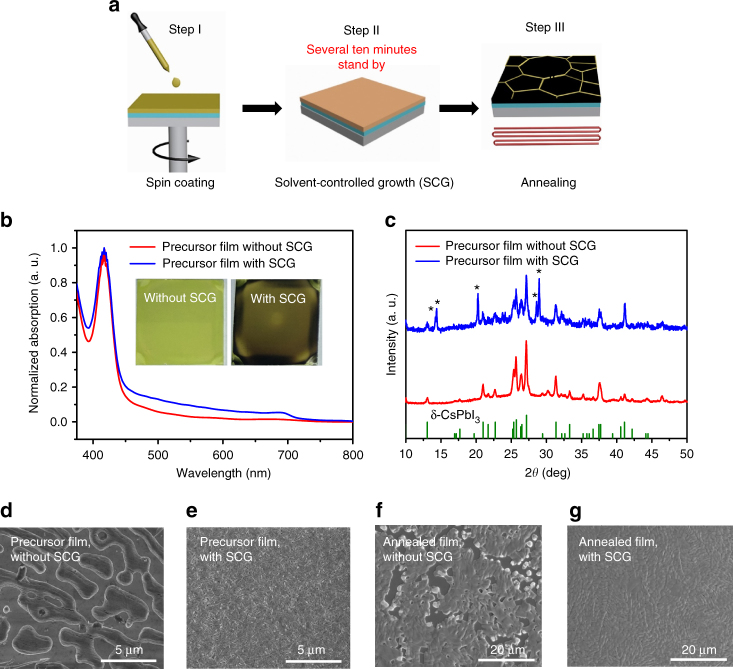
Solvent-controlled growth (SCG) for CsPbI_3_ deposition. **a** Schematic illustration of CsPbI_3_-perovskite crystallization procedures via solvent-controlled growth (SCG). **b** Normalized absorption of CsPbI_3_-precursor films with and without SCG, inset shows the precursor film images without and with SCG. **c** X-ray diffraction (XRD) pattern of CsPbI_3_-precursor films without and with solvent-controlled growth (SCG). Without SCG, the diffraction peaks are mainly from the δ-phase CsPbI_3_, while after SCG, part of δ- phase CsPbI_3_ was transferred into β-phase CsPbI_3_ (a slight distorted α-phase CsPbI_3_). The diffraction peaks labeled as “*” are the diffraction peaks from the β-phase CsPbI_3_. **d**, **e** Scanning electron microscopy (SEM) image of CsPbI_3_ perovskite precursor film without and with SCG, respectively, scale bar: 5 μm. **f**, **g** SEM images of annealed CsPbI_3_ perovskite precursor films without and with SCG, respectively, scale bar: 20 μm

We prepared the CsPbI_3_-precursor films by spin-coating a solution containing PbI_2_ and CsI in a mixture solvent of N,N-dimethylformamide (DMF) and dimethyl sulfoxide (DMSO). It is expected that high boiling point solvent, DMSO (189 °C), could not easily completely escape from the precursor film after spin-coating. The residual DMSO could enhance the mass transport and diffusion, which could improve the film quality if we slow down the evaporation rate of the solvent. Based on this idea, we stand by the precursor films in the nitrogen glove box for several ten minutes before annealing, we named this process as SCG (Fig. 1a).

**Fig. 2:**
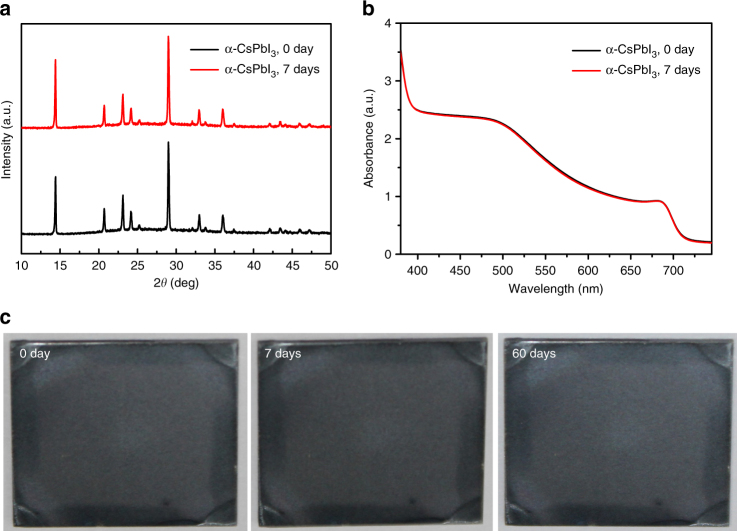
Phase stability of α-CsPbI_3_ films in dry nitrogen environment. **a** X-ray diffraction (XRD) of CsPbI_3_-precursor films annealed at 350 °C for 10 min, all the diffraction peaks from the α-phase of CsPbI_3_, and also the XRD pattern of α-CsPbI_3_ after storing in a dry nitrogen box for 7 days. **b** Absorption of the α-phase of CsPbI_3_ films before and after 7 days of storage in dry nitrogen. **c** Images of annealed CsPbI_3_ films stored in dry nitrogen box for different days

The spin-coated precursor films without SCG showed greenish-yellow color (inset of Fig. 1b, Supplementary Fig. 1). After SCG, we observed that the color of the precursor films gradually changed from greenish-yellow to light black during SCG (inset of Fig. 1b, Supplementary Fig. 1). As a result, in addition to the absorption edge at 460 nm, an absorption in the visible region was observed, indicating that an additional phase has been formed during SCG. The absorption edge of this additional phase is located at 720 nm, which is similar to α-phase CsPbI_3_ (713 nm) (Figs. 1b and 2b), but with a little red-shift. X-ray diffraction (XRD) results further confirmed the appearance of the additional phase after SCG. For the precursor films without SCG, only the diffraction peaks from the δ-phase CsPbI_3_ were observed. While for the precursor films with SCG, except for the diffraction peaks from the δ-phase CsPbI_3_, obvious diffraction peaks located at 14.2°, 14.4°, 20.2°, 28.6°, and 29.0° were observed. These diffraction peaks could be from β-phase CsPbI_3_, which is also a black phase, while owns a slightly distorted crystal structure compared with α-phase CsPbI_3_^49^ (Figs. 1c and 2a, Supplementary Fig. 2b). Similar β-phase CsPbI_3_ has been observed while using phenylethylammonium-stabilized CsPbI_3_ films^49^. The partial phase change from the δ-phase CsPbI_3_ to the β-phase CsPbI_3_ after SCG indicated that the precursor materials were diffused and the precursor film was reconstructed during solvent evaporation. This enhanced mass transport process could be in favor of the uniform and high-quality film formation.

Accomplished with the formation of an additional phase in the precursor film, we also found that the precursor film became more continuous, and the pinholes in the precursor films have been filled after SCG (Fig. 1d, e). The improvement of morphology also indicated the enhanced diffusion and also the mass transportation during SCG. This will be helpful for obtaining high-quality α-phase CsPbI_3_ perovskite film after annealing. Similar SCG method has been adopted in organic solar cells, while high boiling point solvent such as dichlorobenzene is used, and the enhanced polymer crystallization is observed^50^, while the crystal growth kinetic seems different from the SCG methods shown in this study.

As we expected, the annealed CsPbI_3_-perovskite films with SCG are free of pinholes and showed crystal size above 5 μm (Fig. 1g, Supplementary Fig. 3). However, the CsPbI_3_-perovskite films obtained without SCG usually showed a large number of pinholes (Fig. 1f). The morphology evolution dependent on the SCG times can be found in Supplementary Fig. 4. The significant improvement in perovskite films morphology indicated that SCG process is in favor of obtaining high-quality perovskite films.

### Stability of the α-phase CsPbI_3_ film in a dry environment

We carried out the XRD measurement for the annealed CsPbI_3_ films. We found that α-phase CsPbI_3_ crystal was formed by two steps from the precursor films with SCG. For the precursor film with SCG, the δ-phase and β-phase of CsPbI_3_ coexisted at room temperature, and then was completely changed to δ-phase CsPbI_3_ after annealing at 150 °C. After that, the precursor film was completely converted into α-phase CsPbI_3_, while the temperature was increased to 350 °C (Supplementary Fig. 2a). Encouragingly, we found that the high quality of α-phase CsPbI_3_ that we obtained via SCG method can be maintained in dry nitrogen environment for a long time, and there is no change in the XRD patterns or absorption spectra after 7 days of storage (Fig. 2a, b). And furthermore, the films were not degraded even after more than 2 months of storage in a dry environment (Fig. 2c), indicating that the dry environment can freeze the α-phase of CsPbI_3_^25^. Consistent with the previous reports^25, 34, 35^, we also found the induction of phase changes from black to yellowish-white after exposing the films to ambient air with high humidity (Supplementary Fig. 5). Our results showed that the α-phase of CsPbI_3_ can be maintained at room temperature if we can completely avoid moisture, and additional additives were not needed for phase stabilization^25, 40, 41, 43–45^. Therefore, we can absolutely achieve stable CsPbI_3_-based solar cells if we can completely avoid moisture.

### Characterizations of CsPbI_3_ film

We found that the photoluminescence (PL) emission peaks of CsPbI_3_ were blue-shifted from 710 to 703 nm after SCG for 50 min (Supplementary Fig. 6), this could be due to the reduction of defect-related traps, while the film quality was improved^51^. We tested the time-resolved photoluminescence (TRPL) of the CsPbI_3_ films, the lifetime of CsPbI_3_ was increased from 0.6 to 5.2 ns after SCG for 50 min (Supplementary Fig. 6), further confirming that the traps have been reduced after SCG of the perovskite layer. We also found that the PL of CsPbI_3_ was improved gradually with increasing SCG time (Supplementary Fig. 6). The lifetime of SCG CsPbI_3_ films is in few nanoseconds, which is still shorter than that of organic–inorganic hybrid perovskite with the lifetime in microseconds^52, 53^, this could be further improved in future.

We characterized the chemical states and also the band structure of CsPbI_3_ film, the full spectrum of X-ray photoelectron spectroscopy (XPS) and core energy-level spectra confirming the inclusion of Cs, Pb, and I element (Supplementary Fig. 7). And ultraviolet photoelectron spectroscopy (UPS) measurement was also carried out to determine the band structure of CsPbI_3_. It could be estimated that the conduction band and valence band of CsPbI_3_ are about 3.95 eV and 5.68 eV, respectively (Supplementary Fig. 8).

### CsPbI_3_-based solar cells and device performance

We adopted the high-quality SCG-CsPbI_3_ perovskite films as the absorption layer to configure solar cells with the structure of Indium tin oxide (ITO)/SnO_2_/CsPbI_3_/Spiro-OMeTAD/Au. N-type of SnO_2_ was used as the electron transport layer, which was confirmed as an excellent electron transport layer in organic–inorganic perovskite solar cells (Supplementary Fig. 9)^10^, and Spiro-OMeTAD was used as the hole transport layer. A cross-sectional scanning electron microscopy (SEM) image of the completed device is shown in Supplementary Fig. 10. It was found that there is no obvious grain boundary for CsPbI_3_ in about 3 μm scale. From the SEM image, it can be also estimated that the thicknesses of the SnO_2_, perovskite layer, Spiro-OMeTAD, and Au are about 25 nm, 350 nm, 170 nm, and 60 nm, respectively.

**Fig. 3:**
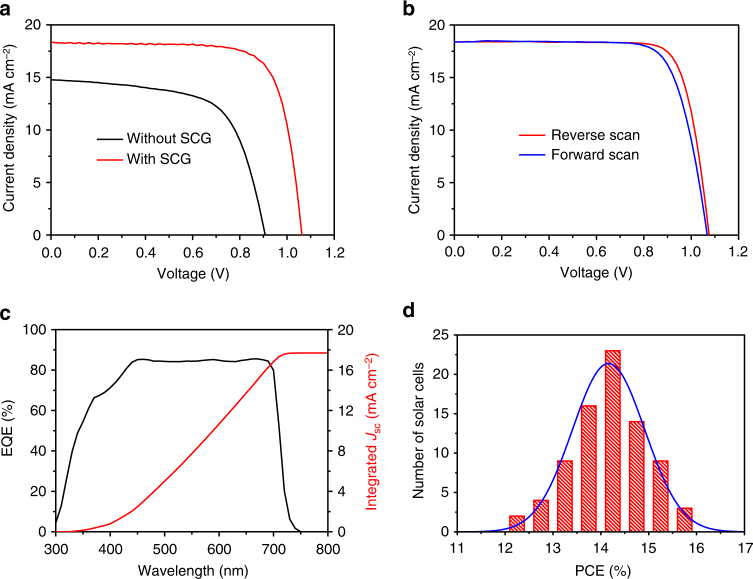
Device performance of CsPbI_3_-based solar cells. **a** *J*–*V* curves of the devices using CsPbI_3_ as the absorber layer, while the CsPbI_3_ without and with solvent-controlled growth (SCG), the SCG time is 50 min. **b** The device performance under forward scan (0 to 1.2 V) and reverse scan (1.2 to 0 V) for the best performance device. **c** External quantum efficiency (EQE) spectrum of a CsPbI_3_ solar cell (black) and the integrated short-circuit current density (red). **d** Device performance distribution for 80 devices, the curve represents the Gaussian function of the histogram

The device performance for the CsPbI_3_ films with and without SCG was characterized (Fig. 3a, Supplementary Fig. 11). Specifically, when the perovskite layer is without SCG, the devices showed poor performance, with open circuit voltage (*V*_OC_) of 0.91 V, short-circuit current density (*J*_SC_) of 14.77 mA cm^−2^, fill factor (FF) of 64%, and the efficiency of only 8.58%. The lower performance might be due to too many pinholes in the perovskite layer and lead to serious leakage and recombination^46^. While SCG were carried out, the device performance enhanced significantly (Fig. 3a, Supplementary Fig. 11 and Supplementary Table 1). The best performance of 15.71% was obtained when the perovskite layer with optimized SCG time for 50 min, with the *V*_OC_ of 1.08 V, *J*_SC_ of 18.41 mA cm^−2^, and FF of 79.32% (Fig. 3b, reverse scan). The efficiency we achieved here represents a great improvement compared with the previous reports in CsPbI_3_-based solar cells (Supplementary Table 2). We tested the device performance under reverse and forward scan, the *J*–*V* curves show no appreciable hysteresis between the two different scan directions (Fig. 3b). Reverse and forward scans showed the efficiency of 15.71% and 14.93%, respectively, the average efficiency is about 15.32% (Fig. 3b). The typical external quantum efficiency (EQE) of the CsPbI_3_ solar cells was given (Fig. 3c); the photoresponse edge is about 720 nm, corresponding to the bandgap of CsPbI_3_ (1.73 eV). In the visible region, the EQE can reach up to 85% with an integrated short-circuit current of 17.7 mA cm^−2^, which is almost consistent with the *J*–*V* result (Fig. 3a, b). Our devices also showed good reproducibility, the efficiency is located from 12.3 to 15.7% for 80 devices, and most of the PCEs are about 14% (Fig. 3d).

We encapsulated the best devices and sent them to photovoltaic calibration laboratory (Newport, an accredited PV calibration laboratory, USA) for certification, which confirmed that a stabilized PCE is 14.67%, the *V*_OC_ is 1.097 V, *J*_SC_ is 18.0 mA cm^−2^, and FF is 74% (Supplementary Figs. 12 and 13). As we know, CsPbI_3_ is very sensitive to moisture, the certificated results showed that there is no significant degradation during shipping and measurements, while the encapsulated devices were completely exposed to ambient air with moisture for a long time (192 h), inferring that the phase stability issue of CsPbI_3_ could be completely solved by encapsulation.

### Device stability

**Fig. 4:**
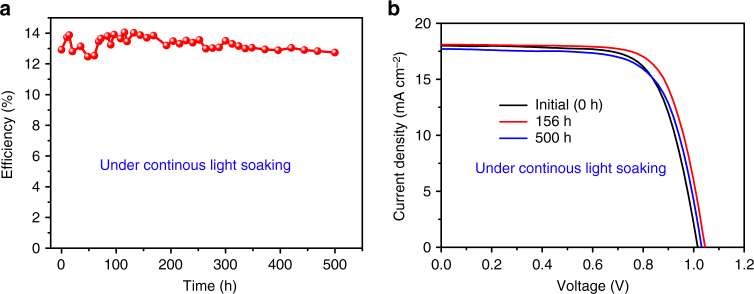
Photostability of the CsPbI_3_ solar cells. **a** Photostability measurement of the devices under continuous one-sun illumination (100 mW cm^−2^) with UV cut filter (420 nm) in nitrogen glove box (temperature: approximately 25 °C) for the unencapsulated devices. **b** *J*–*V* curve of the devices under different continuous light-soaking time

The stability of perovskite solar cells is a critical issue^22, 28, 54–61^, we tested the device's stability in the dark and also under continuous light soaking. We found that the device can almost keep its original efficiency when stored in dry nitrogen for 720 h (30 days) (Supplementary Fig. 14 and Supplementary Table 3). More importantly, we found that our device showed excellent photostability under continuous light soaking in dry nitrogen environment. After 500 h of continuous light soaking (AM 1.5G, 100 mW cm^−2^ with 420 nm UV light filter, temperature: approximately 25 °C), the device can maintain its original efficiency and no drop (Fig. 4a). As an example, the initial PCE of the device is 12.97%, after 156 h of continuous light soaking, the efficiency was slightly increased to 13.7%, which could be due to the improvement of the contact, and then dropped a little to 12.74% after 500 h of light soaking (Fig. 4b, Supplementary Table 4). The good photostability of CsPbI_3_ solar cells could be due to the excellent thermal stability or large ion migration barrier of the inorganic perovskite materials^37^. Longer-time photostability measurements are also carried out. Further improvement of the CsPbI_3_-solar cells stability could be by doping of the perovskite layer^34, 43, 58^, interface engineering^55–57, 59, 60^, and also advanced encapsulation^61^.

## Discussion

We found that our SCG method could be extended to obtain high-quality CsPbI_2_Br films (Supplementary Fig. 15). According to SCG, a 14.21% PCE of CsPbI_2_Br solar cells has been obtained (Supplementary Fig. 16 and Supplementary Table 5). Using this SCG method, we have also achieved as high as 16.14% and 9.81% PCE of CsPb(I_0.85_Br_0.15_)_3_- and CsPbBr_3_-based solar cells, respectively (Supplementary Fig. 17). These results indicated that our SCG method is universal at least for high-quality inorganic perovskite films growth and also for obtaining efficient solar cells.

In conclusion, a 15.7% PCE of CsPbI_3_ solar cells have been achieved by SCG of the absorb layer, and the devices can tolerate above 500 h of continuous light soaking. There is still a large room for device performance, especially on the open-circuit voltage, considering the bandgap of CsPbI_3_ (1.73 eV); a 1.3 V open-circuit voltage should be feasible for CsPbI_3_ solar cells if the contact and the defect can be perfectly controlled, and the efficiency will be close to or beyond 20%.

## Methods

### Materials

SnO_2_-colloid precursor (tin(IV) oxide, 15% in H_2_O colloidal dispersion), DMF, and DMSO were purchased from Alfa Aesar. CsI and PbI_2_ were purchased from Sigma Aldrich.

### Device fabrication

The ITO-coated transparent conducting substrate was successively washed with detergent solution, distilled water, acetone, and isopropanol, respectively. The CsPbI_3_-precursor solution is made by dissolving CsI and PbI_2_ (molar ratio 1:1) in a mixture of DMF and DMSO (v/v, 4:1). A compact 25-nm thin SnO_2_ layer was spin-coated on the glass/ITO substrates, the details could be found elsewhere^10^. And then, the 0.8 M CsPbI_3_-precursor solution was deposited by a one-step spin-coating process onto the transport layer at the speed of 1500 rpm for 45 s. Other compositions of inorganic perovskite films were also used for fabrication of devices to show that our SCG method is a universal approach. For CsPbI_2_Br solution, 0.8 M CsI mixed with 0.4 M PbI_2_ and 0.4 M PbBr_2_ were dissolved in DMF and DMSO solvent. For CsPb(I_0.85_Br_0.15_)_3_ solution, 0.85 M CsPbI_3_ and 0.15 M CsPbBr_3_ were mixed. For CsPbBr_3_ solution, 0.4 M of CsBr:PbBr_2_ (1:1) was dissolved in DMF and DMSO solvent. For SCG of the perovskite layer, we dried the fresh spin-coated perovskite precursor films in a glove box, ranging from 0 to 50 min. After drying, the precursor films were annealed at 350 °C for 10 min in nitrogen glove box for the formation of α-phase CsPbI_3_ (the annealing temperature of CsPbBr_3_ is 250 °C). For conventional growth without SCG, after spin-coating of perovskite precursor films, annealing was carried out immediately. After cooling of the annealed perovskite films, the Spiro-OMeTAD hole transport layer was applied by spin-coating at 2500 rpm for 30 s. A total of 1 mL of Spiro-OMeTAD/chlorobenzene solution contained 72.3 mg Spiro-OMeTAD with the addition of 35 μL lithium bis(trifluoromethanesulphonyl)imide/acetonitrile (260 mg mL^−1^) and 30 μL 4-tert-butylpyridine. Eventually, 60 nm of gold electrode was thermally evaporated on top of the device through a shadow mask, with an effective area of 0.108 cm^2^.

### Characterization

UV–vis spectra were carried out on a Varian Cary 5000 spectrophotometer. SEM measurements were measured with FEI NanoSEM650 to get the relevant parameters, including the morphology and composition of the films, additionally, also includes the device structures. The XRD patterns (θ–2θ scans) were recorded with a Rigaku D/MAX-2500 system operated Cu Kα (*λ* = 1.5405 Å) at 40 kV and 200 mA. Steady PL measurement was carried out by Nanolog TCSPC (USA), TRPL were carried out by Edinburgh Instruments F900 (UK). During XRD and PL measurement, to avoid degradation of the films, the films were spin-coated with PMMA for protection, the concentration of the PMMA solution was 8% in chlorobenzene, and the spin rate was 2000 rpm. UPS measurements were conducted on a Thermo Scientific ESCALab 250Xi using HeI (21.22 eV) radiation lines. XPS were also carried out on the Thermo Scientific ESCALab 250Xi with 200 W monochromated Al Kα (1486.6 eV) radiation, and the XPS analysis using a 500 μm X-ray spot. Current–voltage characteristics of the photovoltaic devices were measured with a Keithley 2400 source meter under a simulated AM 1.5G spectrum and a solar simulator (Enli Tech, Taiwan), before each measurement, the solar simulator was calibrated with a Si solar cell (KG-5). The *I*–*V* measurements were carried out in nitrogen glove box. The devices are both measured in reverse scan (1.2 to 0 V, step 0.02 V) and forward scan (0 to 1.2 V, step 0.02 V), the photovoltaic devices were measured in both forward scan and reverse scan at a scan rate of 0.02 V s^−1^. The devices were taken out for EQE measurement, the EQE were measured by Enli Tech (Taiwan) EQE measurement system. Devices were encapsulated by the UV-epoxy and use-edge encapsulation method. For our best devices, we encapsulated the devices and then sent to PV calibration laboratory (Newport, an accredited PV calibration laboratory, USA) for certification, and during testing, a metal mask with the size of 0.0738 cm^2^ has been used. For shelf-stability test, we stored the solar cells in nitrogen glove box and measured it intermittently, and we collected the shelf-stability in 720 h. For the photostability test, the devices were soaked under continuous one-sun condition with UV cut filter (AM 1.5G, 100 mW cm^−2^, 420 nm cut filter), the *J*–*V* curves were collected every several hours, and we collected the device's photostability in 500 h. The photostability test was also carried out in nitrogen glove box for the device without encapsulation.

### Data availability

The data that support the findings of this study are available from the corresponding author upon reasonable request.

## Electronic supplementary material

Supplementary Information
