# Supplementary material for: Solvent-controlled growth of inorganic perovskite films in dry environment for efficient and stable solar cells
Source: Nat Commun. 2018 Jun 8;9:2225. doi: 10.1038/s41467-018-04636-4 (PMC5993712; doi:10.1038/s41467-018-04636-4)
Supplement: Supplementary file 1 — Supplementary Information [file 41467_2018_4636_MOESM1_ESM.pdf]

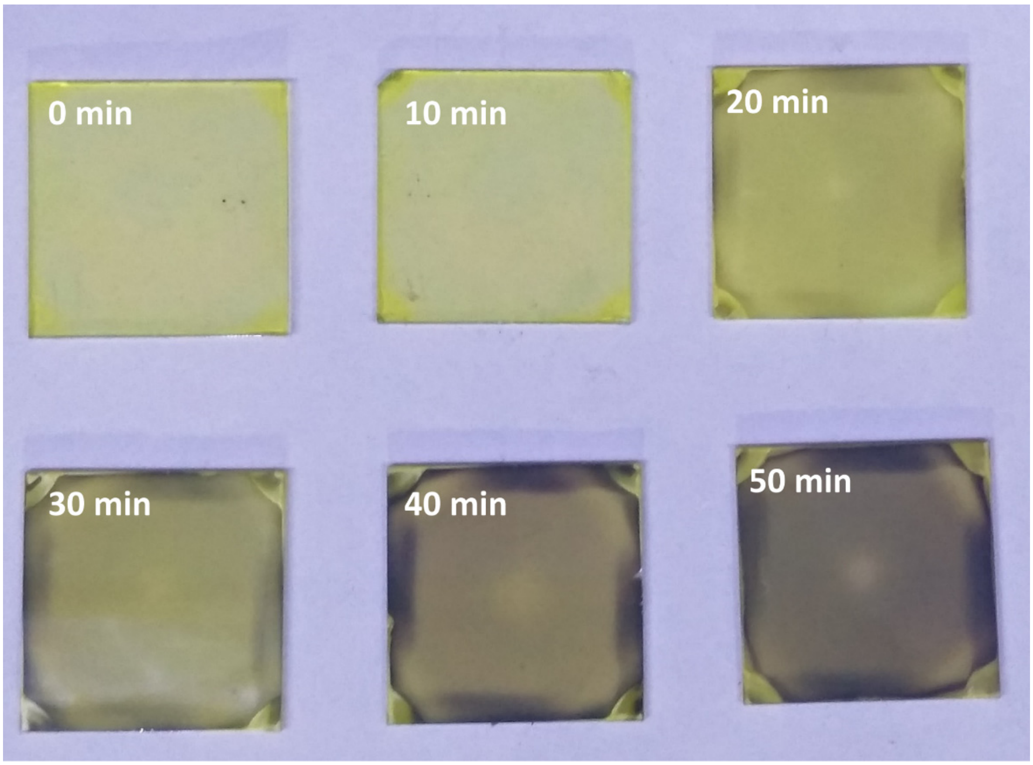


**Supplementary Figure 1. The precursor films changes during the Solvent controlled growth (SCG) from 0 min to 50 min before annealing.** We can see after 20 min SCG, the precursor films gradually become light dark, indicated that a self-organization process happened during the solvent drying.

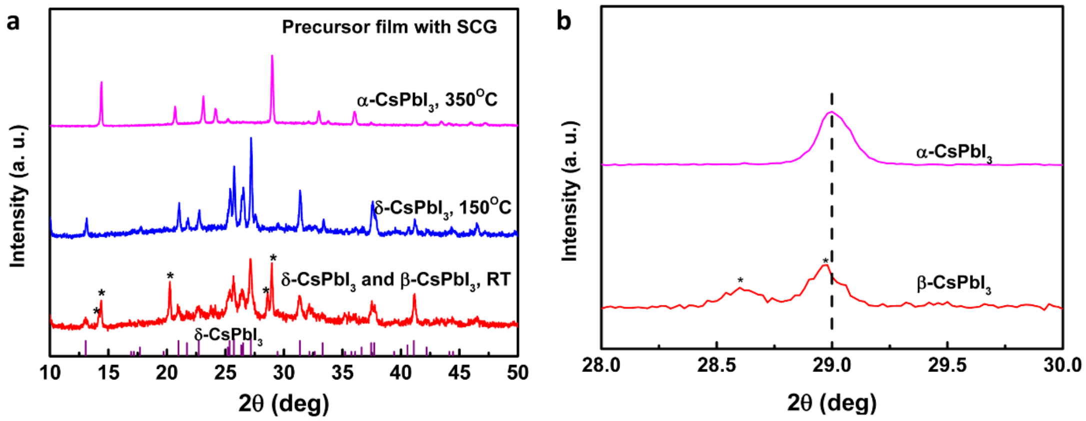


**Supplementary Figure 2. a**, X-ray diffraction (XRD) patterns of the $CsPbI_3$ precursor films with self-organization growth (SCG) annealed at different temperature, it can be found the precursor films experienced two steps before formation of α-phase $CsPbI_3$. At room temperature, the precursor film contains δ-phase and β-phase $CsPbI_3$; when the temperature was increased to 150°C, the main phase in the films are δ-phase; and α-phase can be formed at 350$^{O}$C. **b**, The enlargement of the diffraction angles from 28° to 30° for the RT and 350°C, it can be found that the β-phase $CsPbI_3$ and δ-phase $CsPbI_3$ showed different diffraction angle position.

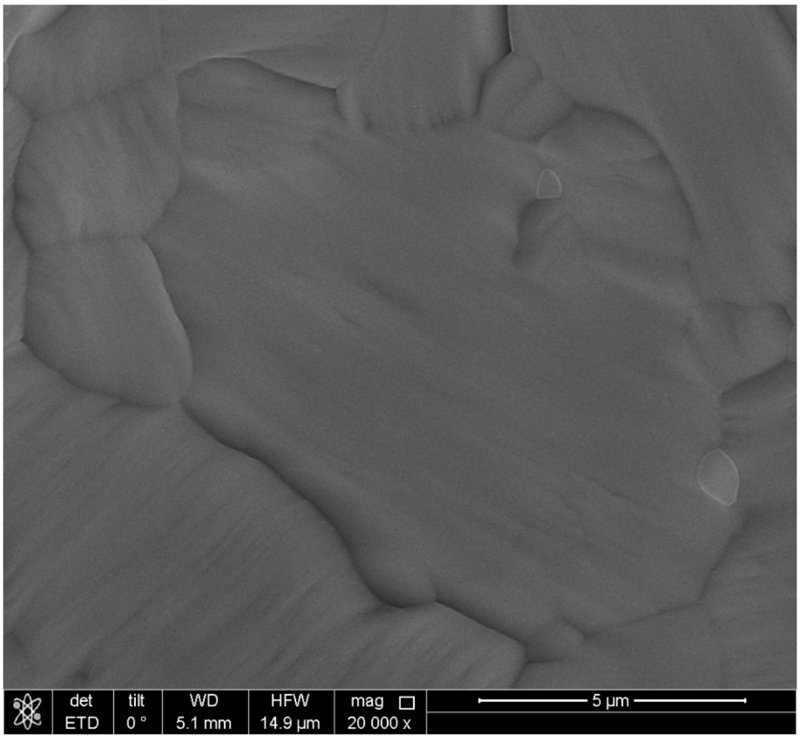


**Supplementary Figure 3**. Enlargement of the scan electron microscopy (SEM) image of solvent controlled growth of $CsPbI_3$ films, scale bar 5 mm.

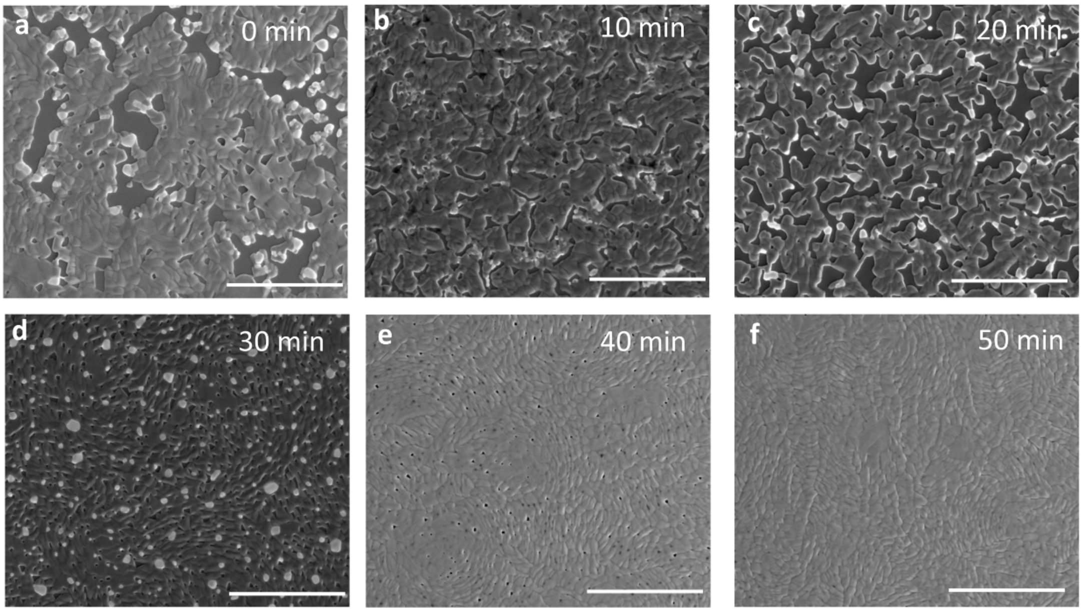


**Supplementary Figure 4**. Morphology evolution of $CsPbI_3$ film with different Solvent controlled growth (SCG) time. **a-f**, 0 min-50 min. The films were annealed in nitrogen glove box at 350°C for 10 min for formation of α-phase $CsPbI_3$. The scale bar are all 20 μm.

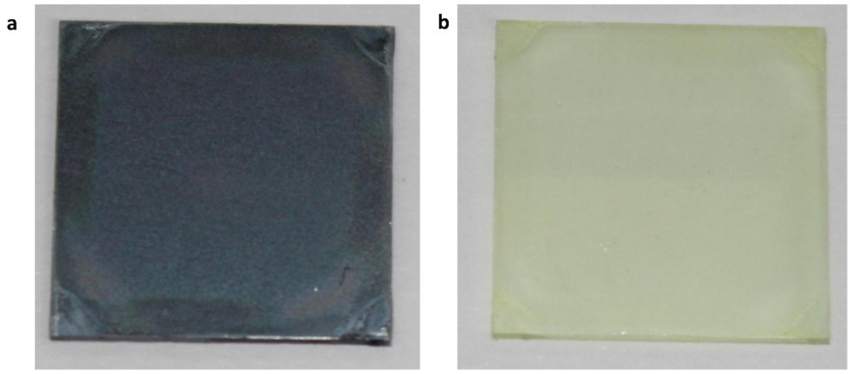

**Supplementary Figure 5. a,** Image of black phase of $CsPbI_3$ formed by annealing the precursor film under dry nitrogen at 350$^{o}$C for 10 min. **b**, Image of the yellow phase of $CsPbI_3$. The yellow phase was formed by exposing the black phase of $CsPbI_3$ shown in supplementary Figure 4a into ambient air with high moisture level overnight.

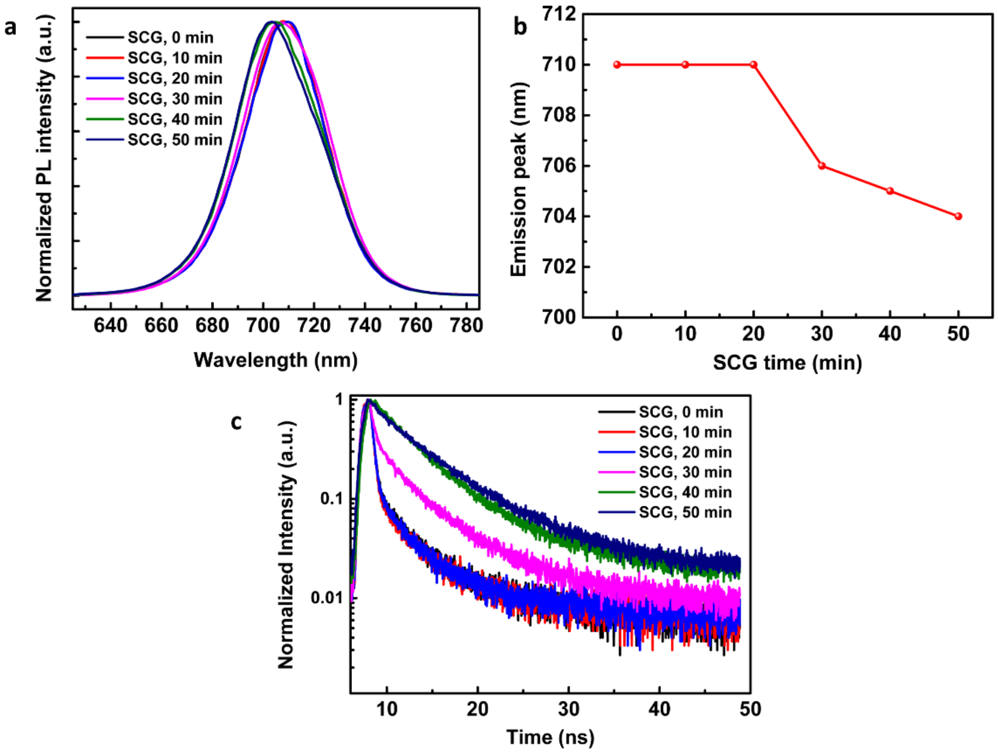


**Supplementary Figure 6.** Emission properties of perovskite films under different Solvent controlled growth (SCG) time. **a**, Steady photoluminescence (PL) of $CsPbI_3$ with SCG ranging from 0 min to 50 min. **b**, The emission peak change with SCG time, the data are collected from supplementary Figure 6a. **c**, Time resolved photoluminescence (TRPL) of $CsPbI_3$ with SCG time ranging from 0 min to 50 min.

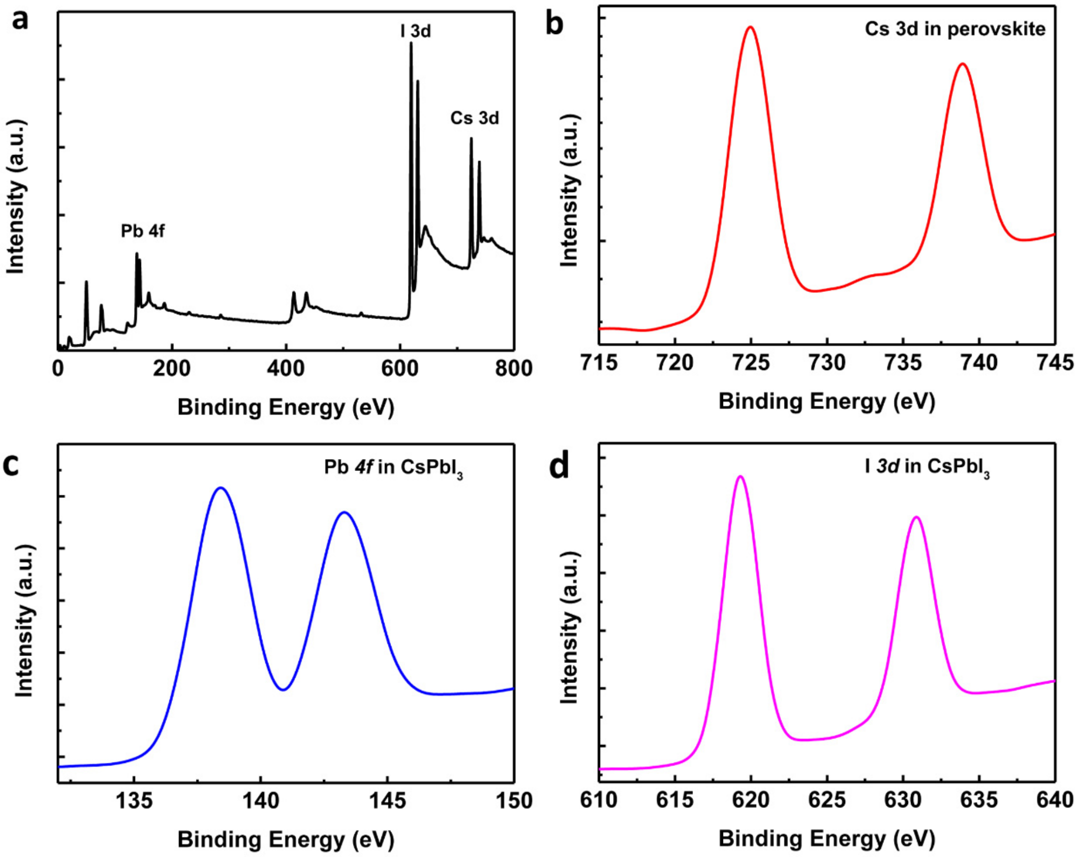


**Supplementary Figure 7**. **a**, X-ray photoelectron spectroscopy (XPS) of $CsPbI_3$ film and also the core energy level of Cs 3d, Pb 4f and I 3d, which were shown in **b**, **c** and **d**, respectively.

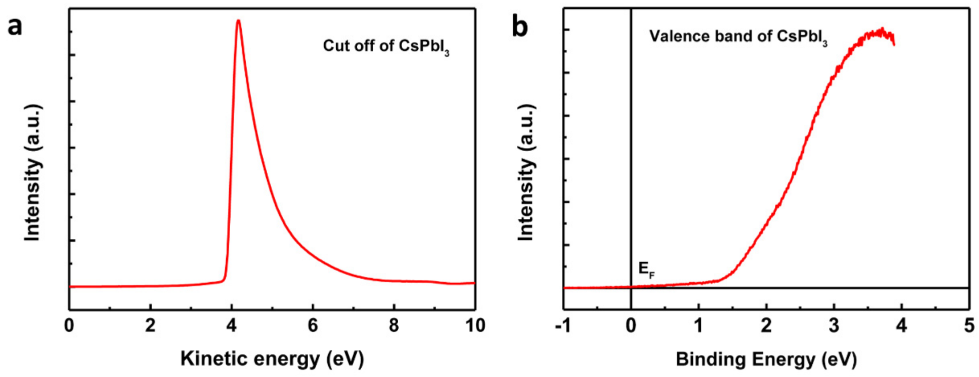


**Supplementary Figure 8.** Ultraviolet photoelectron spectroscopy (UPS) of $CsPbI_3$. **a**, Cut off of $CsPbI_3$, $E_{cutoff}$ of $CsPbI_3$ is 3.89 eV according to the leading edge, the work function ($W_F$) of $CsPbI_3$ can be calculated to be 4.21 eV based on $h\nu$-$W_F$ = $E_{Fermi}$-$E_{cutoff}$, here, hν = 21.22eV, $E_{Fermi}$ = 20.9eV (using Ni as the standard sample for calibration). **b**, The valence band maximum (*VBM*) spectra, the VBM of $CsPbI_3$ is 1. 47 eV according to the distance between leading edge to Fermi energy level ($E_F$), and the valence band $E_V$ ($E_V$=$W_F$+VBM) of $CsPbI_3$ will be 5.68 eV, and the conduction band $E_C$ will be 3.95 eV ($E_c$=$E_v$-$E_g$).

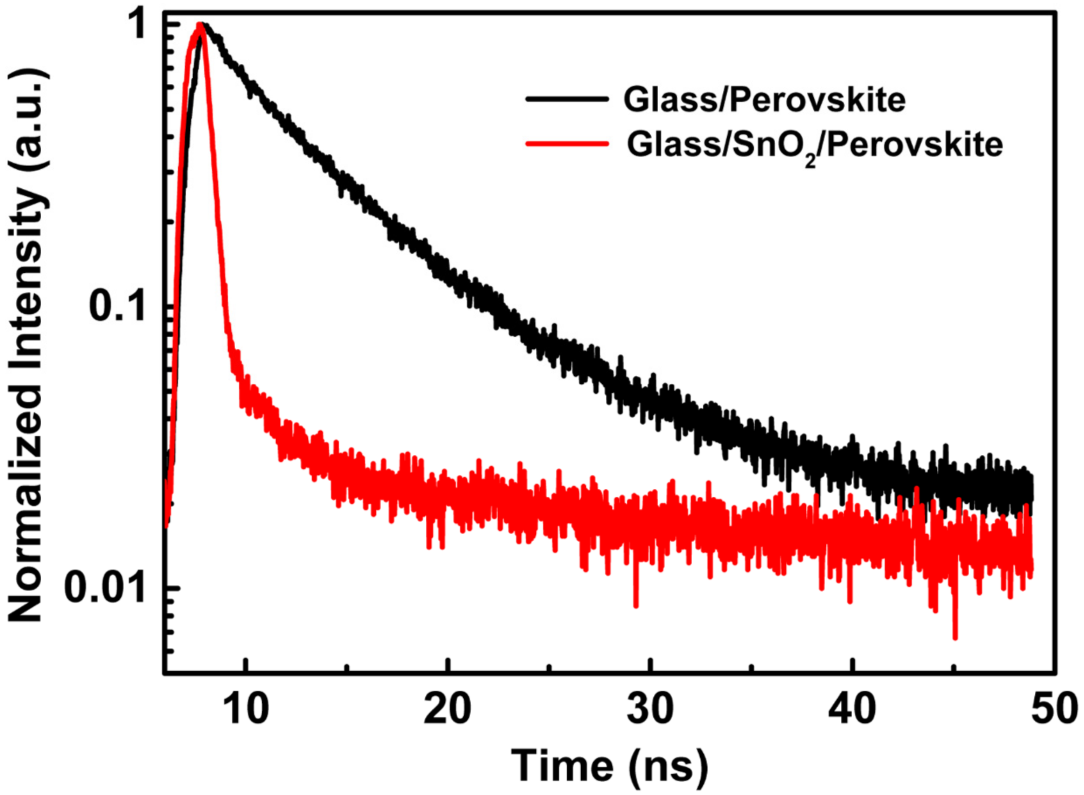


**Supplementary Figure 9.** Time resolved photoluminescence (TRPL) of $CsPbI_3$ film deposited on glass and $SnO_2$ surface, respectively. The decrease of life time indicating an efficient charge transfer happened from $CsPbI_3$ to $SnO_2$ electron transport layer.

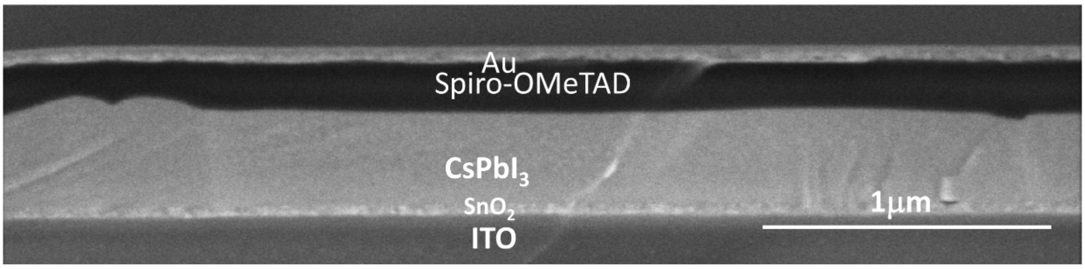


**Supplementary Figure 10**. Cross-section of $CsPbI_3$ solar cells with the structure of Glass/ITO/$SnO_2$/$CsPbI_3$/Spiro-OMeTAD/Au. The thicknesses of $SnO_2$, $CsPbI_3$, Spiro-OMeTAD, Au are 25 nm, 350 nm, 170 nm and 60 nm, respectively.

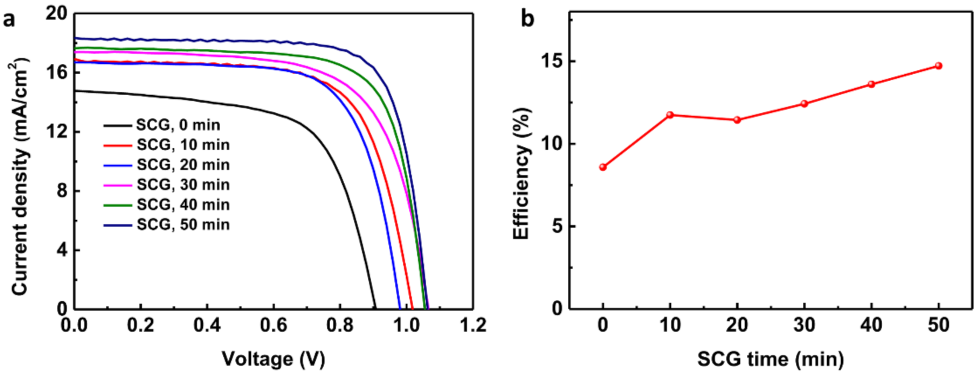


**Supplementary Figure 11. a**, J-V curves of the devices using $CsPbI_3$ as absorber layer, while the $CsPbI_3$ precursor films self-organization growth (SCG) for different times. **b**, Power conversion efficiency depended on SCG time of $CsPbI_3$ layer.

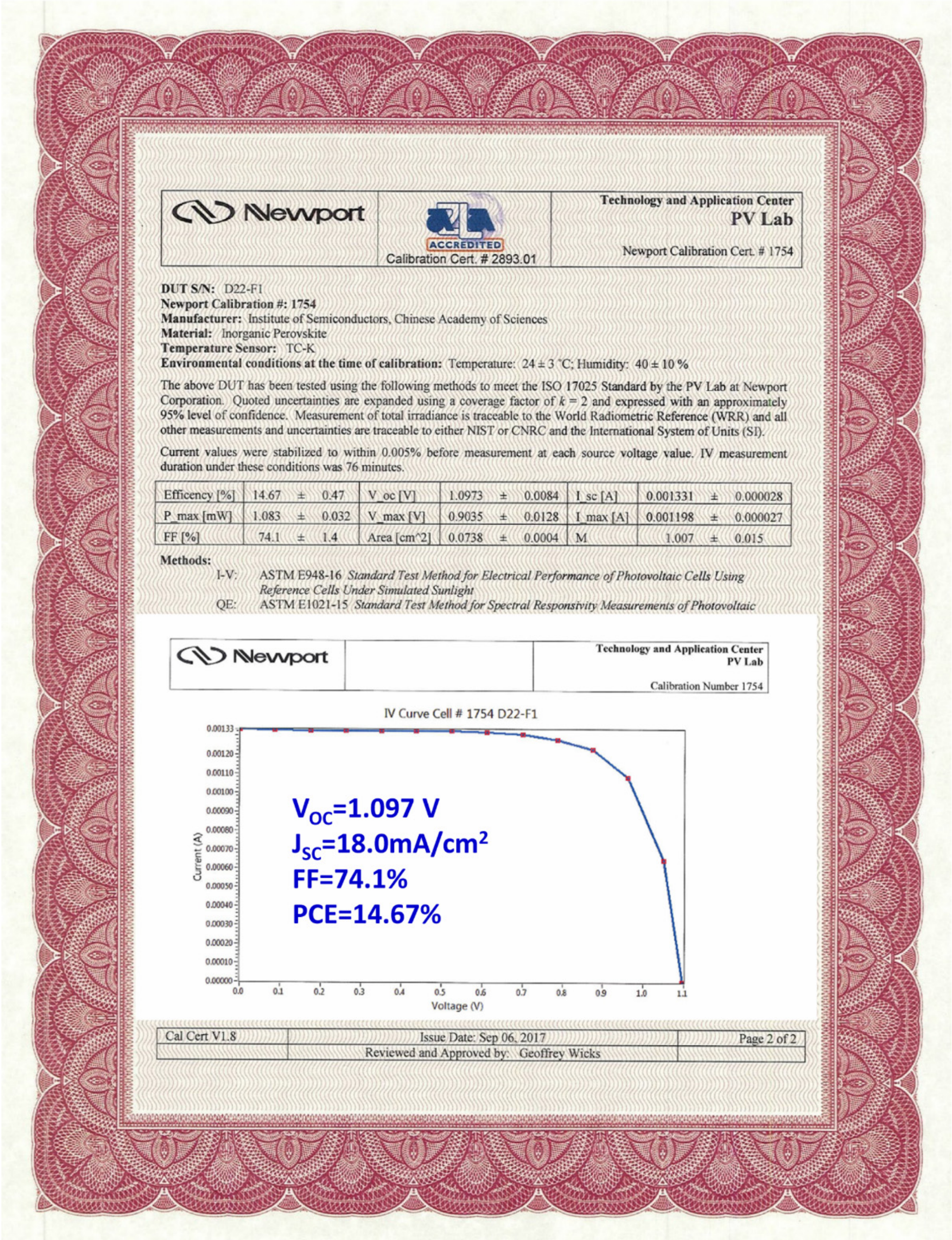

Newport | ACCREDITED Calibration Cert. # 2893.01 | Technology and Application Center PV Lab Newport Calibration Cert. # 1754

**DUT S/N:** D22-F1
**Newport Calibration #: 1754**
**Manufacturer:** Institute of Semiconductors, Chinese Academy of Sciences
**Material:** Inorganic Perovskite
**Temperature Sensor:** TC-K
**Environmental conditions at the time of calibration:** Temperature: 24 ± 3 °C; Humidity: 40 ± 10 %

The above DUT has been tested using the following methods to meet the ISO 17025 Standard by the PV Lab at Newport Corporation. Quoted uncertainties are expanded using a coverage factor of *k* = 2 and expressed with an approximately 95% level of confidence. Measurement of total irradiance is traceable to the World Radiometric Reference (WRR) and all other measurements and uncertainties are traceable to either NIST or CNRC and the International System of Units (SI).

Current values were stabilized to within 0.005% before measurement at each source voltage value. IV measurement duration under these conditions was 76 minutes.

| | | | | | | | | | | | |
|---|---|---|---|---|---|---|---|---|---|---|---|
| Efficency [%] | 14.67 | ± | 0.47 | V_oc [V] | 1.0973 | ± | 0.0084 | I_sc [A] | 0.001331 | ± | 0.000028 |
| P_max [mW] | 1.083 | ± | 0.032 | V_max [V] | 0.9035 | ± | 0.0128 | I_max [A] | 0.001198 | ± | 0.000027 |
| FF [%] | 74.1 | ± | 1.4 | Area [cm^2] | 0.0738 | ± | 0.0004 | M | 1.007 | ± | 0.015 |

**Methods:**
I-V: ASTM E948-16 *Standard Test Method for Electrical Performance of Photovoltaic Cells Using Reference Cells Under Simulated Sunlight*
QE: ASTM E1021-15 *Standard Test Method for Spectral Responsivity Measurements of Photovoltaic*

Newport | Technology and Application Center PV Lab Calibration Number 1754



| | | |
|---|---|---|
| Cal Cert V1.8 | Issue Date: Sep 06, 2017 | Page 2 of 2 |
| | Reviewed and Approved by: Geoffrey Wicks | |

**Supplementary Figure 12. Certificated results of our $CsPbI_3$ solar cells from an accredited photovoltaic certification laboratory (Newport， USA)**. The certificated efficiency is 14.67±0.47%. Our devices area was 0.108 cm$^2$, while certification, a mask with the area of 0.0737 cm$^2$ was used. Inset shows the certificated I-V curve, this *I-V* curve was obtained by stabilizing the device for more than 70 minutes. The $V_{OC}$ is 1.097 V, $J_{SC}$ is 18.0 mA/cm$^2$, FF is 74.1% and PCE is 14.67%.

| | | |
|---|---|---|
| Newport | | **Technology and Application Center**<br>**PV Lab**<br><br>Calibration Number 1754 |

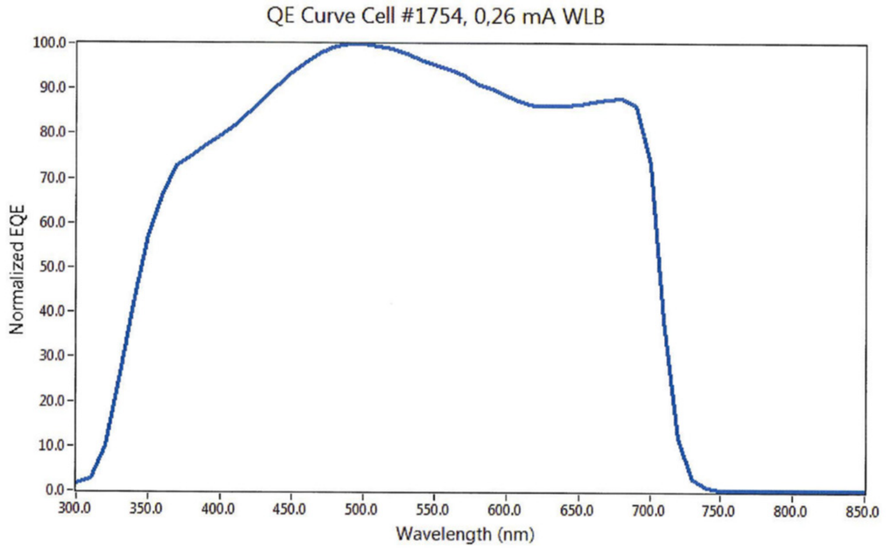


| | | |
|---|---|---|
| Cal Cert Data V1_2 | Issue Date: Sep 06, 2017 | Page 3of 5 |
| | Reviewed and Approved by: Geoffrey Wicks | |

**Supplementary Figure 13**. The external quantum efficiency (EQE) of our $CsPbI_3$ solar cells measured at Newport laboratory in USA.

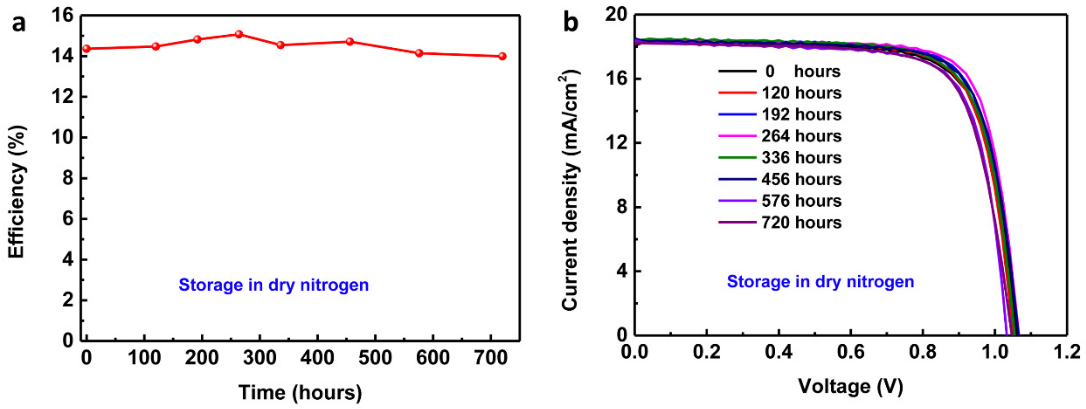


**Supplementary Figure 14.** J-V curve of the devices stored in dry nitrogen glove box without encapsulation for different times. We collected the data for 720 hours.

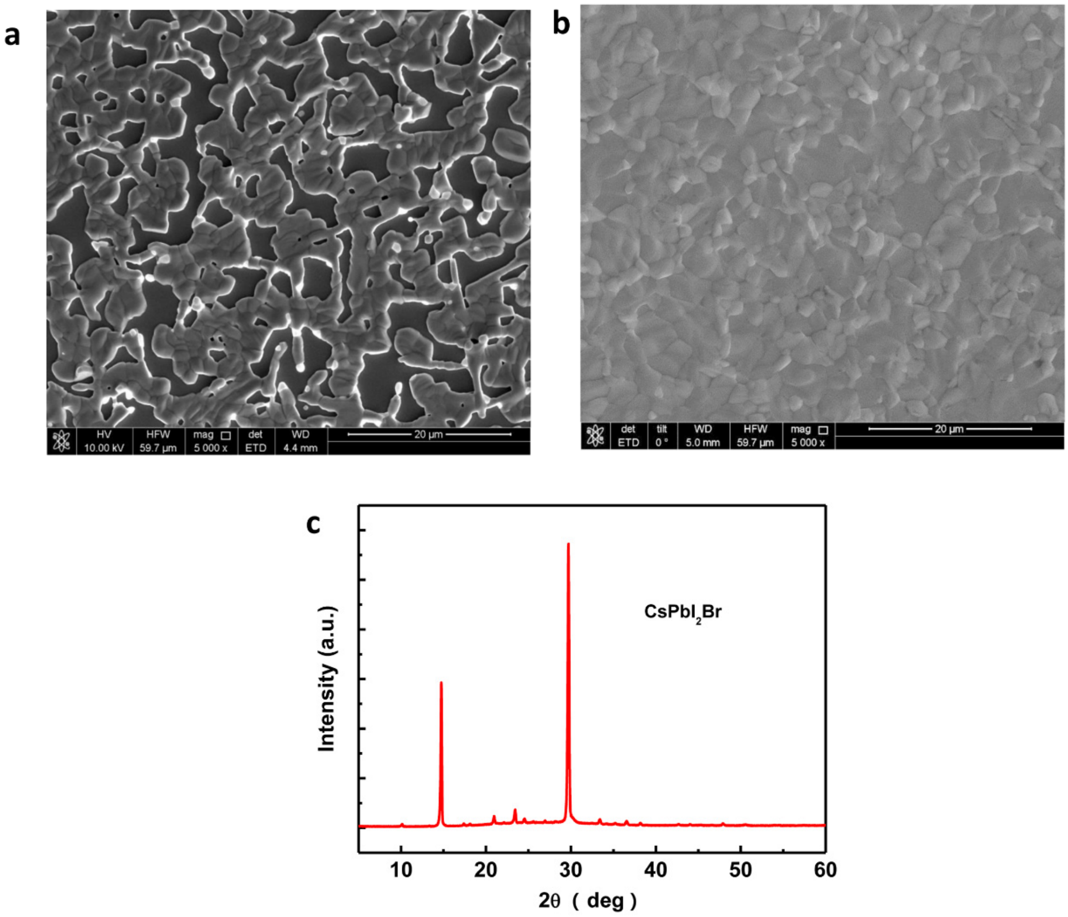


**Supplementary Figure 15.** **a**, Scanning electron microscopy (SEM) image of $CsPbI_2Br$ film without Solvent controlled growth (SCG). **b**, Scanning electron microscopy (SEM) image of $CsPbI_2Br$ film after SCG. **c**, X-ray diffraction (XRD) pattern of $CsPbI_2Br$ film with SCG.

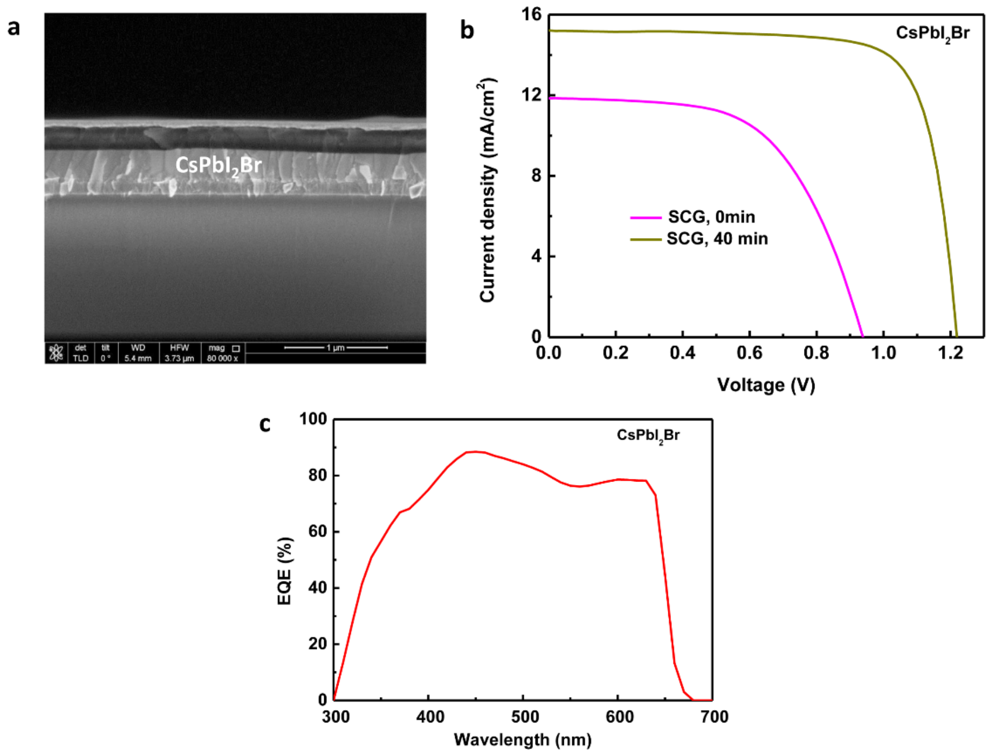


**Supplementary Figure 16**. **a**, Cross-section of $CsPbI_2Br$ based solar cells (with SCG) with the structure of Glass/ITO/$SnO_2$/$CsPbI_2Br$/Spiro-OMeTAD/Au. **b**, J-V curve of the $CsPbI_2Br$ based solar cells with and without self-organization of $CsPbI_2Br$ layer, the performance were summarized in the table S5. **c**, External quantum efficiency (EQE) of $CsPbI_2Br$ solar cells with SCG.

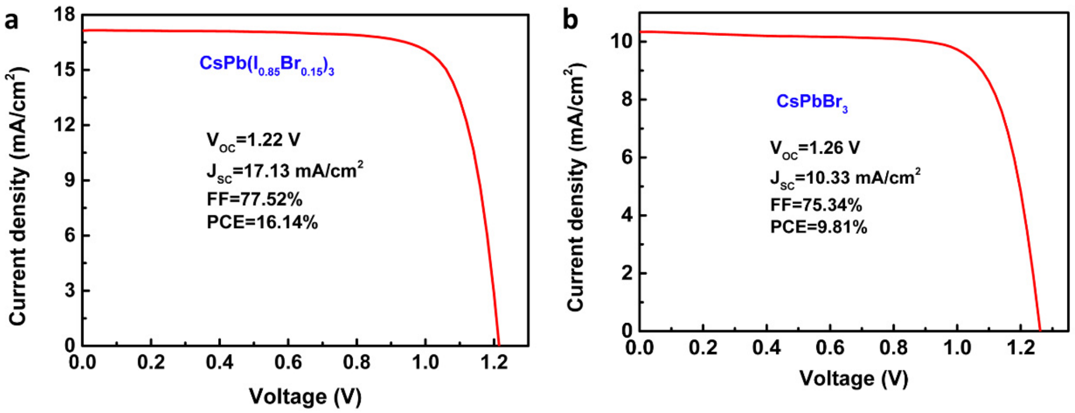


**Supplementary Figure 17.** J-V curve of the $CsPb(I_{0.85}Br_{0.15})_3$ and $CsPbBr_3$ based solar cells using self-organization growth of the absorb layers, as high as 16.14% and 9.81% power conversion efficiency have been obtained for $CsPb(I_{0.85}Br_{0.15})_3$ and $CsPbBr_3$ based solar cells.

**Supplementary Table 1.** Summary of the device performance while $CsPbI_3$ precursor films via solvent controlled growth (SCG) for different time ranging from 0 min to 50 min.

| SCG time (min) | $V_{OC}$ (V) | $J_{SC}$ (mA cm$^{-2}$) | *FF* (%) | *PCE* (%) |
|---|---|---|---|---|
| 0 min | 0.91 | 14.77 | 63.97 | 8.58 |
| 10 min | 1.02 | 16.93 | 68.08 | 11.74 |
| 20 min | 0.98 | 16.69 | 69.86 | 11.44 |
| 30 min | 1.07 | 17.39 | 67.00 | 12.42 |
| 40 min | 1.06 | 17.64 | 73.05 | 13.60 |
| 50 min | 1.06 | 18.35 | 75.40 | 14.72 |
| **50min（best）** | **1.08** | **18.41** | **79.32** | **15.71** |

**Supplementary table 2**. Summary of the best-reported of $CsPbX_3$ (X=Br, I or their mixture) solar cells.

| Perovskite materials | Deposition methods and Processing | Experimental Environment | PCE (%) | Ref. |
|---|---|---|---|---|
| $CsPbI_3$ | Solution process, SCG | Nitrogen | **15.71** | **This work** |
| $CsPbI_3$ | Thermal deposition | Air-free | 10.5 | [36] |
| $CsPbI_3$ | Solution process, adding sulfobetaine zwitterions | Nitrogen | 11.4 | [38] |
| $CsPbI_3$ | HI additive and IPA treatment | Open air | 4.13 | [41] |
| EDA-$CsPbI_3$ | EDA-stabilized $CsPbI_3$ | Nitrogen | 11.8 | [42] |
| FA-CsPbI3 | FA-passivated $CsPbI_3$ | Nitrogen | 13.43 | [43] |
| Bi-$CsPbI_3$ | Bi doped $CsPbI_3$ | Nitrogen | 13.21 | [41] |
| $CsPbI_3$ QDs | Solution process | Open air | 10.77 | [40] |
| $CsPbI_2Br$ | Solution process, SCG | Nitrogen | **14.21** | **This work** |
| $CsPb(I_{0.85}Br_{0.15})_3$ | Solution process, SCG | Nitrogen | **16.14** | **This work** |
| $CsPbI_2Br$ | Thermal deposition | Air-free | 11.8 | [37] |
| $CsPbI_2Br$ | Solution process | Nitrogen | 9.8 | [33] |
| $CsPbBr_3$ | Solution process, SCG | Nitrogen | **9.81** | **This work** |
| $CsPbBr_3$ | Solution process | Open air | 5.95 | [19] |

**Supplementary table 3**. Device performance of the devices stored in dry nitrogen glove box without encapsulation for different times.

| **Storage time (hours)** | $V_{OC}$ **(V)** | $J_{SC}$ **(mA cm$^{-2}$)** | ***FF*** **(%)** | ***PCE*** **(%)** |
|---|---|---|---|---|
| 0 | 1.06 | 18.08 | 75.00 | 14.36 |
| 120 | 1.05 | 18.22 | 75.58 | 14.47 |
| 192 | 1.06 | 18.46 | 76.08 | 14.82 |
| 264 | 1.07 | 18.18 | 77.72 | 15.07 |
| 336 | 1.05 | 18.32 | 75.28 | 14.54 |
| 456 | 1.07 | 18.51 | 74.58 | 14.71 |
| 576 | 1.03 | 18.42 | 74.31 | 14.14 |
| 720 | 1.05 | 18.23 | 73.60 | 14.04 |

**Supplementary table 4**. Device performance of the devices soaking under one sun condition (AM1.5G, 100 mW $cm^{-2}$ with 420 nm UV cut filter) for different times. We have collected the data for 500 hours.

| **Light-soaking time (hours)** | $V_{OC}$ **(V)** | $J_{SC}$ **(mA $cm^{-2}$)** | ***FF*** **(%)** | ***PCE*** **(%)** |
|---|---|---|---|---|
| 0 | 1.02 | 18.00 | 70.61 | 12.92 |
| 14 | 1.03 | 18.68 | 72.13 | 13.87 |
| 20 | 1.00 | 18.28 | 69.82 | 12.81 |
| 35 | 1.01 | 18.00 | 72.18 | 13.14 |
| 48 | 0.993 | 18.55 | 67.67 | 12.47 |
| 60 | 1.00 | 18.36 | 68.08 | 12.54 |
| 68 | 1.03 | 18.60 | 70.59 | 13.46 |
| 72 | 1.02 | 18.79 | 71.00 | 13.65 |
| 84 | 1.03 | 18.87 | 71.22 | 13.81 |
| 90 | 1.02 | 18.31 | 71.12 | 13.25 |
| 96 | 1.03 | 18.91 | 71.78 | 13.91 |
| 108 | 1.02 | 18.47 | 72.39 | 13.65 |
| 115 | 1.04 | 18.52 | 73.17 | 14.05 |
| 120 | 1.03 | 18.23 | 71.91 | 13.46 |
| 132 | 1.05 | 18.24 | 72.82 | 14.02 |
| 144 | 1.04 | 18.48 | 72.46 | 13.87 |
| 156 | 1.05 | 18.00 | 72.49 | 13.70 |
| 168 | 1.04 | 18.54 | 72.11 | 13.84 |
| 192 | 1.01 | 18.14 | 69.30 | 12.73 |
| 204 | 1.04 | 18.31 | 70.56 | 13.48 |
| 210 | 1.04 | 18.13 | 70.68 | 13.31 |
| 218 | 1.05 | 18.34 | 70.38 | 13.52 |
| 240 | 1.05 | 18.09 | 70.72 | 13.38 |
| 252 | 1.04 | 18.43 | 70.77 | 13.56 |
| 264 | 1.04 | 17.91 | 70.06 | 12.99 |
| 276 | 1.04 | 18.46 | 68.17 | 13.03 |
| 288 | 1.03 | 17.84 | 70.81 | 13.07 |
| 300 | 1.04 | 18.67 | 69.58 | 13.50 |
| 312 | 1.04 | 18.19 | 70.54 | 13.30 |
| 324 | 1.04 | 17.95 | 70.56 | 13.16 |
| 336 | 1.03 | 17.90 | 70.34 | 13.00 |
| 348 | 1.04 | 17.92 | 70.25 | 13.05 |
| 372 | 1.03 | 18.06 | 69.43 | 12.94 |
| 396 | 1.03 | 17.84 | 69.99 | 12.89 |
| 420 | 1.03 | 18.00 | 69.91 | 13.02 |
| 444 | 1.03 | 17.94 | 69.66 | 12.90 |
| 468 | 1.03 | 17.82 | 69.75 | 12.83 |
| 500 | 1.03 | 17.70 | 69.74 | 12.74 |

**Supplementary Table 5**. Device performance of the $CsPbI_2Br$ devices with and without solvent-controlled growth (SCG) under one sun condition (AM1.5G, 100 mW $cm^{-2}$).

| **Devices** | ***V*****OC** **(V)** | ***J*****SC** **(mA cm⁻²)** | ***FF*** **(%)** | ***PCE*** **(%)** |
|---|---|---|---|---|
| Without SCG | 0.94 | 11.85 | 57.64 | 6.43 |
| With SCG | 1.22 | 15.22 | 76.58 | 14.21 |
